# ROLE MODEL OF AGING AND ATTITUDES TOWARD AGING IN ASIAN AMERICANS

**DOI:** 10.1093/geroni/igz038.2640

**Published:** 2019-11-08

**Authors:** Seojung Jung, Daniela Jopp

**Affiliations:** 1 SUNY at Old Westbury, Old Westbury, New York, United States; 3 University of Lausanne, Lausanne, Switzerland, Switzerland

## Abstract

While having a role model for successful aging may have a beneficial influence and promote successful aging throughout the course of one’s life, very little empirical research has examined role models for successful aging, particularly among Asian Americans. The current study examined Asian Americans’ role models for successful aging and their link with attitudes toward own aging with a sample of 135 individuals aged 18 to 74 years (Mage = 36.76, SD = 14.73; female, 71.9%). Participants were asked whether they had a specific role model for successful aging and, if they had, the reasons why the role models were chosen. Participants’ open-ended answers were then analyzed using qualitative content analysis using a coding scheme drawn from a prior study on role models for successful aging (Jopp et al., 2016). The categories most often mentioned were public figures, followed by parents, other relatives, non-specific persons, grandparents, non-family acquaintances, and self. Participants with at least one family role model were more likely to have positive attitudes toward aging, and this effect was strong even after controlling for ethnic identity, filial obligation, self-rated health, and other demographic variables. The present study suggests that Asian Americans identify role models for successful aging both within their immediate families and among public figures, while family role models are more likely to promote the development of positive attitudes toward own aging. The implications for understanding Asian Americans’ role models on motivating, promoting, and facilitating positive aging processes will be discussed.

